# RNA-Binding Proteins as Epigenetic Regulators of Brain Functions and Their Involvement in Neurodegeneration

**DOI:** 10.3390/ijms232314622

**Published:** 2022-11-23

**Authors:** Carlo Maria Di Liegro, Gabriella Schiera, Giuseppe Schirò, Italia Di Liegro

**Affiliations:** 1 Department of Biological, Chemical and Pharmaceutical Sciences and Technologies (Dipartimento di Scienze e Tecnologie Biologiche, Chimiche e Farmaceutiche) (STEBICEF), University of Palermo, 90128 Palermo, Italy; 2 Department of Biomedicine, Neurosciences and Advanced Diagnostics (Dipartimento di Biomedicina, Neuroscienze e Diagnostica Avanzata) (Bi.N.D.), University of Palermo, 90127 Palermo, Italy

**Keywords:** post-transcriptional regulation of gene expression, RNA-binding proteins (RBPs), intrinsically disordered regions (IDRs), EVs, learning, memory, synaptic plasticity, neurodegeneration

## Abstract

A central aspect of nervous system development and function is the post-transcriptional regulation of mRNA fate, which implies time- and site-dependent translation, in response to cues originating from cell-to-cell crosstalk. Such events are fundamental for the establishment of brain cell asymmetry, as well as of long-lasting modifications of synapses (long-term potentiation: LTP), responsible for learning, memory, and higher cognitive functions. Post-transcriptional regulation is in turn dependent on RNA-binding proteins that, by recognizing and binding brief RNA sequences, base modifications, or secondary/tertiary structures, are able to control maturation, localization, stability, and translation of the transcripts. Notably, most RBPs contain intrinsically disordered regions (IDRs) that are thought to be involved in the formation of membrane-less structures, probably due to liquid–liquid phase separation (LLPS). Such structures are evidenced as a variety of granules that contain proteins and different classes of RNAs. The other side of the peculiar properties of IDRs is, however, that, under altered cellular conditions, they are also prone to form aggregates, as observed in neurodegeneration. Interestingly, RBPs, as part of both normal and aggregated complexes, are also able to enter extracellular vesicles (EVs), and in doing so, they can also reach cells other than those that produced them.

## 1. Introduction

For many years, most of the studies aimed at understanding the regulation of gene expression were devoted to the analysis of DNA–protein interactions involved in the structural organization of chromatin of both repressed and active genes, and in the process of transcription. It is now clear, however, that, once transcribed, RNA faces many different steps of maturation and traffic that can deeply modify its actual fate, and hence the outcome of gene expression. Such events are most important during development, but also in adult organisms. In particular, they are at the heart of the nervous system’s development and function, both for the establishment of brain cell asymmetry and for nerve cell plasticity and synapse potentiation, processes that are also involved in learning, memory, and higher cognitive functions.

As in the case of the DNA–protein interactions that regulate transcription, RNA destiny strictly depends on nucleic acid–protein complexes, and, in particular, on RNA-binding proteins (RBPs) [1,2,3,4,5,6,7,8,9,10]. As for many other properties of RNA, we can hypothesize that its ability to interact with proteins that regulate its expression is a present-day remnant of an early-life biochemical “RNA world” in which genetic information was stored in RNA molecules [11,12,13,14,15,16,17,18]. It has been suggested, indeed, that, at the beginning of such a primeval world, RNA was able not only to store genetic information and to self-replicate but even to function as a catalyst. This latter hypothesis found an important basis in the discovery of ribozymes and other RNA-catalyzed reactions [19,20,21,22,23,24]. Recently, it has been also suggested that the catalytic power of RNA might have been enhanced by “globularity”, that is by the formation of secondary/tertiary structured domains, and by modified versions of some bases (that might have resembled cofactors) [25,26]. In the last decades, the acknowledgment of RNA versatility further increased after discovering, besides ribosomal and transfer RNAs, a high number of regulatory, non-coding species, among which long non-coding RNAs (LncRNAs), sometimes circularized RNAs (circRNAs), and microRNAs (miRNAs) [27].

It has been also suggested that the coexistence of self-replicating and catalytic RNA with amino acids might have allowed the formation of the first peptides, perhaps constituted by only a few amino acids, such as glycine (Gly/G), alanine (Ala/A), aspartate (Asp/D), and valine (Val/V) [28,29,30,31]. Now, it has been suggested that amyloids may be formed from short peptides showing simple amino acid composition and alternating hydrophobic and hydrophilic residues, in the presence of other simple molecules, such as carbonyl sulfide [32], thus allowing us to hypothesize a primeval “RNA-prion world” or “amyloid world” [32,33,34,35,36,37]. Of course, as we discuss below, amyloids are fibrillary protein aggregates, containing arrays of beta-strands, which accumulate in neurodegenerative pathologies, among which Alzheimer’s and prion diseases. Notably, present-day amyloids can interact with lipids and cause modification of membrane properties, among which its fluidity [32,38,39,40]. Moreover, because of their repetitive structure, amyloids might be able to bind RNA, with a stabilizing effect [41]. Importantly, a variety of experiments suggests that compartmentalization due to lipid complexes, such as coacervates, may have allowed the concentration and self-assembly of RNA molecules, thus enhancing their polymerization and catalytic activities [42,43].

**Figure 1:**
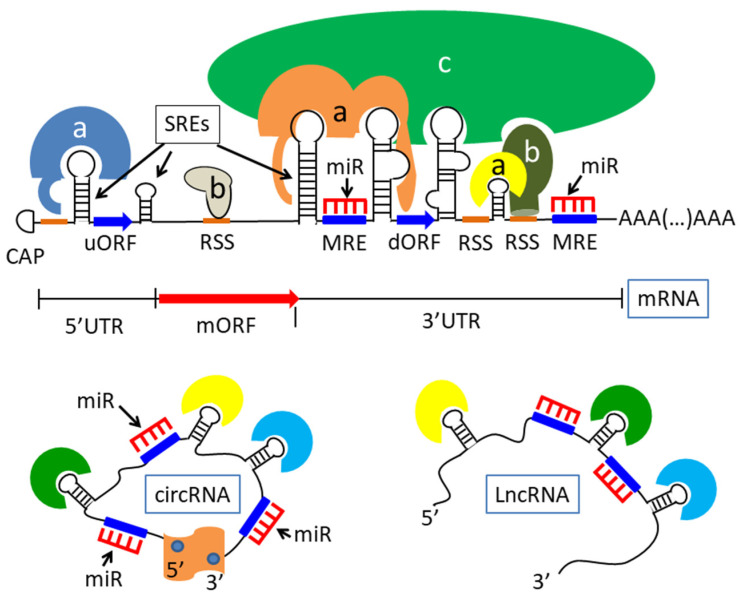
Schematic representation of RBP interaction with either mRNA or with some classes of non-coding RNAs. The mRNA (upper picture) is typically modified at its 5′-end by a cap and at its 3′-end by a poly(A) tail. All mRNAs contain at least one open reading frame (ORF), which is normally translated into protein; this main ORF (mORF) can be modified by cytoplasmatic splicing events, by translation starting at different start codons, or by RNA editing. In addition, mRNA can also contain additional short ORFs in its 5′-untranslated region (5′-UTR) or in its 3′-UTR (upstream ORF, uORF, and downstream ORF, dORF, respectively); mRNAs also contain a variety of structural elements (SREs), and recognition simple sequences (RSS, orange segments), recognized and bound by different families of RBPs. Herein, three kinds of proteins that can be found in ribonucleoprotein particles are depicted: (a) RBPs that recognize SREs; (b) RBPs that recognize RSS; and (c) proteins that do not interact directly with RNA but with RBPs. Finally, mRNA contains short miRNA recognition elements (MRE, blue segments), able to pair with short single-strand RNAs, named miRNAs (miR, red). Interestingly, MREs are also present on non-coding RNAs, such as long non-coding RNAs (LncRNAs) and circular RNAs (circRNAs), which can thus function as sponges for miRNAs.

RNA is a highly versatile molecule, able to form a variety of secondary and tertiary structures, based on both Watson–Crick and non-Watson–Crick interactions. Moreover, as mentioned, many classes of RNA do exist, and they continuously crosstalk to each other, thus regulating the actual outcome of gene expression [44,45,46]. For example, it is now widely accepted that mRNA stability and its chance to be translated can be negatively regulated by microRNAs (miRNAs); these latter molecules are short RNAs of about 20 nucleotides, derived from longer precursors, which, in the context of an RNA–protein complex called the RNA-induced silencing complex (RISC), also contain the RBPs known as Argonaute (Ago) proteins and pair with short sequences (six-eight nucleotides), called miRNA response elements (MREs), present for most in the 3′-untranslated region (3′-UTR) of their target transcripts. Each mRNA can contain multiple MREs and, on the other hand, each miRNA can target a high number of mRNAs [47,48]. However, the ability of miRNAs to bind mRNAs is counteracted by both linear and circular long non-coding RNAs (LncRNAs; circLncRNAs), which contain MREs as well and are thus able to function as sponges for miRNAs, and, consequently, to weaken the miRNA effect on mRNA stability and protein synthesis [49,50,51]. As a whole, these observations suggest the existence of an RNA network that, based on both RNA–RNA and RNA–protein interactions, maintains gene expression homeostasis [44,45,46] (Figure 1).

Notably, protein-coding genes can also encode ncRNAs, and, on the other hand, ncRNAs can also contain short open reading frames (ORFs) that are often conserved in evolution, and that, under certain conditions, are translated [27,52]. It is well known since long ago that eukaryotic protein-coding transcripts themselves can undergo alternative maturation, giving rise to different mRNAs, which either encode different isoforms of the same protein, with different properties, or have alternative untranslated regions with a different potential to bind regulatory molecules (RNAs or RBPs) [53,54,55]. Similarly, alternative polyadenylation can have an effect on the sequence of the 3′-UTR and, consequently, on the post-transcriptional regulation of the mRNA potential [55,56,57,58,59,60]. In addition, non-canonical splicing events, among which back-splicing and trans-splicing, are also possible, and can generate either circRNAs or chimeric RNAs, respectively [61]. Notably, although splicing events are normally confined to the nucleus and precede the mRNA transfer to the cytoplasm and its successive localization, retained introns in cytoplasmic RNAs have been found, and this finding, together with the presence in dendrites of proteins involved in splicing, suggests the existence of peripheral splicing events [62,63,64], possibly related to synapse remodeling. Finally, many laboratories reported the presence of RNA nucleotide modifications, such as cytosine [65,66] and adenosine [67,68,69] methylation; these base modifications can be involved in regulating mRNA transport and localization, as well as translation. For example, some mRNAs can be translated in a cap-independent way, and it has been reported that this event, mediated by an “internal ribosome entry site” (IRES), can be activated by binding of N6-methyladenosine (m6A), present in the 5′-UTR, to the translation pre-initiation complex [69]. The discovery of RNA modifications and their impact on transcript metabolism has even prompted researchers to talk about “epitranscriptomics” [65,66,70]. Interestingly, the reversible methylation of bases is not the only RNA modification; post-transcriptional modification can be also obtained by RNA editing, catalyzed by adenosine deaminases (ADARs), which deaminates adenosine to inosine, and Apoliprotein B mRNA editing enzyme (APOBEC), which transforms cytosine to uracil [71,72,73]. All these processes are certainly involved in modulating mRNA interaction with RBPs. All the steps of mRNA synthesis, splicing, transport, localization, and translation are indeed controlled by a variety of RBP families. Herein, we review a collection of studies that have shown the involvement of many RBPs both in the physiology and pathology of brain cells.

## 2. Post-Transcriptional Regulation of Gene Expression in the Nervous System

The post-transcriptional control of gene expression has a fundamental role in tissue development and differentiation, and is mainly realized thanks to cis-acting sequences present in the RNA itself, usually in the 5′- or 3′-UTR, and by a set of RBPs able to recognize those sequences (Figure 1). In the nervous system, where the pre-localization of the messengers and local translation are involved in axon and dendrite branching and pathfinding, and in synapse formation, post-transcriptional regulation assumes an even greater importance. Notably, local translation may stably modify synapse structure and activity, opening the way to long-lasting modifications in neuronal connections that even support learning and memory capacities [74].

A typical feature of the post-transcriptional regulation is the presence in the cells of specific tridimensional structures that carry both mRNA molecules and regulating proteins. Once transcribed, indeed, messengers are subjected to splicing and polyadenylation, and are eventually moved to the cytoplasm as components of ribonucleoprotein complexes (RNPs), often referred to as granules, many kinds of which have been described [75]. Actually, in the cells, messengers probably exist exclusively inside these specialized complexes, wherein some proteins directly bind RNAs (Figure 1, proteins indicated as “a” and “b”), while others are part of the RNPs because they interact with each other, forming oligomers (Figure 1, proteins indicated as “c”) [8,76].

### 2.1. Mechanisms of Controlled RNA Metabolism, Localization, and Translation

Post-transcriptional control actually includes multiple steps of regulation, some of which have been studied in detail (i.e., alternative splicing, RNA localization, and local translation), while others, such as RNA editing and alternative polyadenylation, still remain quite elusive [8]. In particular, polyadenylation seems to have a double-fold role in the metabolism of mRNA, because the length and composition of the 3′-UTR depend on the site of the poly(A) tail addition, and because mRNA’s half-life and its translatability are influenced by the length of the poly(A) [8,55,57,59,60]. Moreover, during the maturation of the 3′-UTR, a tissue-specific mechanism named alternative cleavage and polyadenylation (APA) may create different versions of the mRNA endowed with specific characteristics of regulation [77]. As mentioned above, the direct modification of the transcripts, by modification of the nucleotides, is also possible and gives rise to what has been called ‘epitranscriptomics’ [65,67,69,70,78]. The most studied of them, involved in the control of translation, concerns N6-methyladenosine (m^6^A), a reversible modification depending on the activity of two classes of enzymes: methyltransferases, which add methyl groups and are thus named ‘writers’, and demethylases called ‘erasers’. The modification may be recognized in specific conditions by a group of RNA-binding proteins acting as ‘readers’ [68]. It has been demonstrated that this mode of regulation participates in the local control of protein synthesis in axons [79].

One main step during the production of mature RNAs is the excision from the messengers of the introns. Splicing is a quite complex mechanism directed by sequences present on the mRNA, which are processed by a set of proteins and small RNAs grouped in small nuclear ribonucleoproteins (snRNPs). Different RBPs may bind mRNA sequences to help reach the conformational structure necessary for intron excision, thus facilitating, or impeding, the inclusion of specific exons [80,81,82,83]. The choice of alternative exons allows the production of different protein isoforms, but also can change the regulatory elements located in the 3′-UTR, thus modifying both the final localization of the messengers and the timing of their translation. For example, BDNF and CaMKIIα mRNA isoforms bearing 3′-UTRs, which are shorter and lack dendritic targeting elements (DTEs), do not reach dendrites [8]. Interestingly, local splicing has been suggested to occur in dendrites of living neurons, indicating local control of 3′-UTR diversity [62]. A good example of the effect of alternative splicing is given by the activity of CaMKIIβ variants in developing neurons bearing the translation product of exon E1: only these molecules, indeed, may associate with F-actin in microspikes of the arborizing dendrites in developing neurons [84].

A striking example of the complexity of splicing is offered by neurexins. These latter proteins act as presynaptic receptors for molecules located on the other side of the synapse, the neuroligins. Thanks to a very complex transcriptional and post-transcriptional control, including a nearly cell-specific splicing program, many different neurexin isoforms are produced in the brain; actually, these molecules are able to act as receptors for several binding proteins, suggesting that they can behave like a core for the organization of the presynaptic space [85,86,87]. Therefore, the complex alternative splicing of neurexin mRNAs is most probably carried out by cell-type-specific RBPs that endow different neuronal cell types with a peculiar Neurexin isoform that, in turn, can react with a set of specific receptors in the post-synaptic cell, contributing to the conference of a specific behavior to a given neuron [86,87].

Interestingly, some intron sequences, found in cytoplasmic mRNAs, act as DTEs, since they are sufficient to target messengers to dendrites. As mentioned, indeed, spliceosome elements and RBPs involved in splicing were identified in the cytoplasm, and more specifically, in dendrites themselves, raising the possibility that splicing could also be carried out in unusual cytoplasmic sites [62,88]. Such ‘dendritic spliceosomes’ could function as a secondary activator of ‘cytoplasmic intron-retaining transcripts’ (CIRTs) that are in a silent state because they are not completely spliced [64].

On the other hand, RBPs may control their own number by mechanisms that reduce their mRNA abundance, via, for example, nonsense-mediated mRNA decay (NMD), a cytoplasmic process that causes the degradation of messengers bearing premature termination codons (PTCs) [89]. It has been demonstrated that these codons can be inserted in the mRNAs by alternative splicing, resulting in their rapid turnover [90]. Thus, translation-dependent NMD seems to be a critical regulator of splicing factors. In line with this notion is the finding that Pumilio proteins, known regulators of RNA stability and translation, regulate the expression of Nova2 [66]. Nova proteins regulate more than 700 alternative splicing events in vivo [91]. Importantly, Nova-controlled splicing mainly affects synaptic proteins [91], such as Gephyrin (Gphn), the scaffold protein for inhibitory synapses [92]. These findings suggest that Nova proteins control neuronal excitation by regulating the inhibitory pathway. For several other splicing factors, such as RNA-binding protein fox-1 homolog (Rbfox1), and Polypyrimidine tract-binding protein (Ptbp) 1 and 2, a similar mechanism has been observed [81]. Consequently, the combination of different RBPs bound to a particular pre-mRNA decides whether an exon is spliced out or not [66].

**Figure 2:**
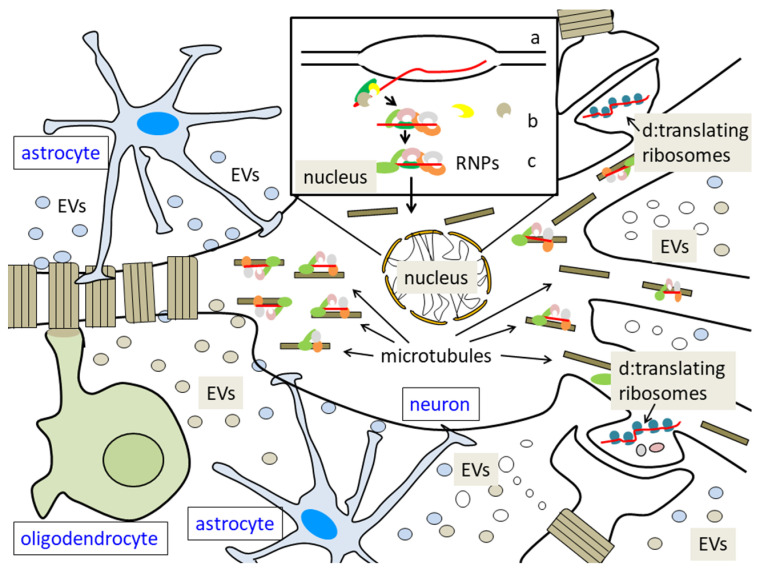
Schematic drawing of mRNA maturation, transport to both dendrites and axon, and translation. As shown in the insert, nascent RNA starts interacting with RBPs already during transcription (a); after its synthesis, heterogeneous nuclear RNA faces splicing: during this phase, some more proteins bind to it (b); after splicing, some RBPs detach, while new ones, probably involved in mRNA transport, bind (c). Finally, mature RNA, enclosed in ribonucleoparticles (RNPs), is ready for transport to the cytoplasm. Once in the cytoplasm, RNPs interact with microtubules and are transported to the periphery where, in response to specific signals, mRNAs are translated (d). Some RNPs can also enter EVs, the extracellular structures released by all cell types of the nervous system; EVs can mediate exchange of many different molecules: metabolites and lipids, but also proteins and RNA of different classes, among which mRNAs, miRNAs, and LncRNAs.

Given the early binding of RBPs to nascent mRNAs, transcripts leave the nucleus as ribonucleoproteins (RNPs) (Figure 2); how these latter complexes pass through the nuclear envelope is not completely clear, but they probably follow the same route that other molecules and complexes do, i.e., nuclear pores, but an unusual way of transfer has also been described, assuming the possibility that they may ‘bud’ through the nuclear membrane [93]. In any case, the next necessary step is transport, to localize mRNAs in specific regions of the cells. Despite the great difference between the low number of known RBPs involved in the transport and the high number of messengers so far identified in neuritis, it has been suggested that each RNP granule might contain single copies of specific mRNA [94,95]. However, other observations suggest that more RNA species may be contained in the same RNPs. The coordinated expression of neural genes may be obtained, indeed, by the assembly of their mRNAs in common mRNP complexes, which, under different cell states and at different times, can contain different RBPs. This process is of particular importance in modulating synaptic plasticity, which seems to depend on the activity-induced translation of many locally targeted mRNAs present in neuronal processes [96].

An example of a very specific RNA–RBP interaction is the transport of β-actin mRNA by ZBP1 RBP. This interaction seems to be crucial to processes such as migration and differentiation, because it allows the localized translation just where active actin polymerization is needed [97]. In the granules containing ZBP1, translation is inhibited by repressors, suggesting that protein synthesis is halted while transport occurs [98]. The transport of β-actin mRNA by ZBP1-containing granules is allowed by the interaction with the Kinesin family member 11 (Kif11) of motor proteins [99]. Translation repression during transport has also been demonstrated in the case of granules containing Staufen and the fragile X mental retardation protein (FMRP) [66]. The FMRP seems to bind mainly at coding regions of the mRNAs, preferentially at TGGA sequences [100]. Its mode of action includes the inhibition of messengers by masking them in granules [101], the block of ribosomal activity [102], and the inhibition of elongation factor eIF4E and eIF4G interaction [103]. Moreover, FMRP has also been suggested to be part of a control system for the regulation, during early neuronal development, of the transition from neural stem cells to intermediate progenitors, differentiation, and migration, together with components such as N-cadherin [104]. Another way to inhibit translation by the FMRP is obtained in association with the RNA-Induced Silencing Complex (RISC). FMRP binding has been recognized as an important contribution to the capacity of the component of the complex to bind and repress mRNA utilization [105,106,107,108].

As long as it concerns Staufen (Stau), transport mediated by this protein is based on microtubule activity, as well as kinesin motors [109]. In the mammalian brain, Stau-mediated transport, in some cases, is dependent on the presence, in mRNAs, of specific introns retained during splicing; for example, only Calmodulin3 (Calm3) mRNA isoform with a longer 3′-UTR and the CaMKII mRNA isoform, which retains intron 16, can reach dendrites by binding Stau2 [8,110].

Concerning calmodulin, an interesting observation made some years ago in our laboratory suggests that the calmodulin-binding, brain-specific Pep19/Pcp4 peptide might play a role in the regulation of the translation of some RNAs; we found, indeed, that this peptide is an RNA-binding protein, but its binding activity is in competition with calmodulin [111].

It has also been reported that the cytoplasmic polyadenylation element-binding protein (CPEB) is involved in bidirectional CaMKII mRNA transport in dendrites, in association with kinesin and dynein motors, as well as with Microtubule-associated protein 2 (Map2) [112]. The CPEB is also involved, in the hippocampus, in the transport of the mRNA encoding the brain-derived neurotrophic factor (BDNF), which bears a specific dendritic targeting element in its 3′-UTR. In the dendritic transport of this mRNA, the protein Translin also seems to be involved [113,114]. After transport along microtubules, RNPs can be transferred to the peripheral actin cytoskeleton, thus penetrating the dendritic spines; in this transfer, Fused in Sarcoma (FUS) RBP can be involved, thanks to its ability to interact with the myosin-Va motor protein [115]. In order to explain the recruitment of RNPs to synapses, it has also been suggested that molecules/conditions, generated by synapse activation, may attract them to synapses while they are moving along neuritis by bidirectional transport (sushi-belt model) [4,116].

All the previously described steps aim at mRNA localization, an undoubtedly advantageous process, especially for complex cells such as neurons; it allows, indeed: (i) to rapidly increase locally the amount of a given protein, without the delay that would result from its transport, (ii) to avoid producing proteins in erroneous sites, and (iii) to add a step in the regulation of translation, which may be directly controlled by local signaling [117,118,119].

Interestingly, the study of neuronal somata and extensions using ribo-sequencing, which allows the quantification of actively translated transcripts, revealed more than 800 different mRNAs in dendrites and axons [120,121]. The mRNA localization and translation in axons provides a specific pool of proteins synthesized in situ that may independently regulate regeneration processes and all the mechanisms aimed at elaborating neurotransmitter release [119]. The local coordinated translation of different mRNAs, which constitute a sort of post-transcriptional operon, in response to the specific needs of the cell in any given moment, offers a higher level of control [122].

In an evolutionary perspective, local translation can be considered a way to put part of the regulation of gene expression under the control of elements located at the cell periphery, that is, a way to ‘decentralize’ the control [123].

Fundamental processes in neuron development are elongation of the axon and branch formation, which imply deep modifications in cytoskeleton organization and the participation of actin remodeling enzymes and regulatory proteins. For example, it has been shown in *X. laevis* that β-actin mRNA is locally translated in axons [124]. Different proteins are involved in the process: Vg1RBP, a ZBP1 homolog, transports β-actin mRNA up to the growth cone; then, β-actin can be produced in an asymmetric fashion, under the control of Netrin-1 and BDNF, allowing the turning of the growth cone [125].

Clearly, the possibility to synthesize new proteins locally is of fundamental importance in any step of neuron development, so ribosomes must be present in developing axons and dendrites to ensure peptide production in response to intra- and extra-cellular signals [120,126,127]. One interesting point is that the presence on the place of both ribosomes and mitochondria also allows local production of mitochondrial proteins, contributing to the in situ turnover of the organelles to meet the needs of the process structurally and energetically [128,129].

As discussed below, an ever-increasing amount of data witnesses that the localized regulation of specific mRNA translation, through modification of synaptic activity, is the key for attaining higher brain cognitive capacities, including learning and memory formation [3,120,130]. Notably, localized translation is most probably not limited to neurons but is active also in glial cells, such as oligodendrocytes and astrocytes [131,132].

RBPs also interact with circRNAs and LncRNAs, which appear especially represented in brain cells and possess an independent type of regulation [133]. Many LncRNAs seem to have a role in chromatin organization, probably by their capacity to interact with chromatin-modifying proteins [134], thus forming complex structures in which many different proteins may be included [135,136].

Finally, when talking about RNP trafficking, the recent discovery of a role of lysosomes as further vehicles for granules is also of note. This process seems to depend on the lysosome-associated membrane glycoprotein 1 (LAMP1) and on the granule-associated phosphoinositide-binding protein Annexin A11 (ANXA11) [137]. On the other hand, lysosomes and the process of autophagy are also involved in the stress-dependent degradation of the granule components [138].

### 2.2. Intrinsically Disordered Regions (IDRs)

The study of IDRs is still in its infancy, and details of the interactions between them are scarce, but some hints are already emerging, especially for RBPs, most of which seem to contain IDRs, in addition to RNA-binding domains [139,140,141]. Two peculiar characteristics regarding IDRs are their ‘fuzzy’ binding to their targets [142,143] and their ability to allow proteins to enter granules by liquid–liquid phase separation (LLPS) [144,145,146]. IDRs are characterized by low amino acid complexity (LC), absence of hydrophobic residues, and enrichment in charged residues [147]. The regions with a low amino acid complexity (low-complexity domains, LCDs) may contain poly-glutamine and poly-alanine tracts and seem to favor the structuration of ribonucleoprotein complexes [148,149,150].

Interestingly, a class of IDRs is represented by prion-like domains (PLDs), frequently found in RBPs, such as TAR DNA-binding protein 43 (TDP-43) and FUS. PLDs are enriched in polar residues, but do not contain charged amino acids; moreover, they contain aromatic residues that, together with their disordered structure, are probably determinant for the ability to form and/or enter RNP granules [147,151,152]. It has also been suggested that PLDs probably influence the polarity of the mRNAs and the diffusion of the ribosomes inside the RNA/protein complexes [153].

Notably, while studying the interaction between HnRNP-A2 and TDP-43, a mechanism of reciprocal regulation through IDRs was evidenced: in some neurodegenerative diseases (see Section 4), TDP-43 shows a transition from an alfa-helix to a beta-sheet structure, which favors its aggregation, and it was found that an increase in the disordered conformation of HnRNP-A2 is directly related to the increase in the beta-sheet structure in TDP-43 [154]. Such a kind of interaction, based on IDRs, is probably responsible for the aggregation-dependent alterations noticed in most neurodegenerative diseases.

Due to their disordered structure, IDRs could mediate the transient interactions required for granule formation [147]. Actually, this latter phenomenon has recently been directly connected with synapse ontogenesis and activity [155,156], and with long-term memory (LTM) consolidation [147]. From this point of view, a particularly interesting protein with prion-like domains seems to be the already mentioned CPEB protein, indeed required for LTM [147]. One interesting possibility is that the secondary structure of the included RNAs is what would also (or even primarily) regulate the stabilization of the RBP structure, thus allowing protein binding and assembly of the granules, which are then involved in RNA transport, localization, and translation [157].

### 2.3. Brain Cell Asymmetry

Localization in dendrites of the mRNAs encoding MAP2 [158] and CaMKIIα [159] opened the way to discover a connection between localization and translation of given messengers in specific regions of neurons, and synaptic activation. Besides these mRNAs, other intensively studied messages localized in dendrites are those encoding BDNF, the activity-regulated cytoskeleton-associated protein (Arc), the NMDAR NR1 subunit, and the AMPA receptor [160,161]. Such mRNAs contain, in their 3′-UTRs, a DTE that is necessary for specific trafficking. Shorter 3′-UTRs that result from alternative splicing and miss the DTE impede their correct positioning [162,163,164,165,166]. Actually, in nearly all analyzed mRNAs, the 3′-UTR is the region on which their localization depends [167], because it contains the so-called zipcodes [117], specific signals that interact with different RBPs [168], creating various kinds of RNA–protein complexes [98,169,170]. Even though the mechanisms that lead to the anchoring and eventual translation of the RNAs are less well known [171], it is thought that, once a given activated synapse is reached, the RNPs disassemble, in order to allow translation [95]; thus, novel proteins are produced that can participate in synapse remodeling. In summary, mRNA destination and activity are governed by a sort of ‘RNA signature’ present in its sequence, which allows the recognition and binding of a specific group of regulating RBPs [66,116].

In particular, it was, for example, demonstrated that the localization and translation of the mRNA encoding CaMKIIα depend on the 3′-UTR and are stimulated by synaptic activation [172,173]. The main CaMKIIα activity consists in the phosphorylation of different proteins involved in synaptic plasticity [174], and the deletion of CaMKIIα 3′-UTR affects learning and memory formation. In mutant mice bearing a gene in which the localization sequence has been altered, the mRNA does not reach the dendrites, and CaMKIIα is reduced in the postsynaptic densities (PSDs). Consequently, both spatial and object recognition memory are affected in the mutant animals, dramatically showing the importance of local translation for synaptic plasticity [175].

BDNF has a fundamental role in brain cells, as it is involved in neuronal development and survival. The structuration and function of mature synapses is influenced by local translation depending on BDNF–TrkB signaling. Trk receptors are indeed widespread both in the presynaptic and postsynaptic density of the dendritic spines of cortical neurons [114]. For a long time, BDNF neurotrophin was supposed to be synthesized just in the cell body, then transported along the axon and secreted. Nowadays, it is acknowledged that BDNF can also be synthesized in the presynaptic compartment, participating in the control of axon development [176]. BDNF induces the dephosphorylation of the FMRP, through a mechanism depending on Calcineurin. In this way, BDNF enhances the translation of a set of transcripts binding to the FMRP (reviewed in [114]). BDNF mRNA is also transported to dendrites, and some authors reported a role for Translin in the process [113]. Different transcripts derive from the Bdnf gene, each of which possesses specific 5′- and 3′-UTR and localizes in distinct sites in the cell. In addition, the 3′-UTR contains two polyadenylation sites, so that each transcript has a long- and a short-3′-UTR version. In hippocampal neurons, the 3′-UTR presents some elements necessary to reach dendrites and to bind CPEB1 [177]. The distribution of the BDNF transcripts seems to be regulated by a ‘spatial code’, retaining some variants in the neuronal body, while sending others to the proximal or distal dendrites [178]. BDNF mRNA 3′-UTR is also the target of the well-known neuronal RBP HuD, which stabilizes the BDNF transcripts with the long 3′-UTR and enhances their translation [8,179].

Another well-described factor whose RNA is localized in dendrites is Arc. The corresponding gene is classified as an immediate early one (IEG), because it is turned on by synaptic activation [163]. Arc is involved at various steps in the synaptic signaling, such as the remodeling of the actin cytoskeleton and endocytosis of AMPA receptors [180]. The involvement of Arc in the process of memory formation and maintenance is witnessed by the effect showed in mice by its mutation [181]. Notably, Arc mRNA contains two intronic sequences in its 3′-UTR, one of which allows recycling of the Arc messenger by NMD [182]. Moreover, Arc is probably translated only at specific times in dendrites, and its translation is inhibited by a complex constituted by FMRP [183] and the cytoplasmic FMR1-interacting protein 1 (Cyfip1); this latter binds the 5′-cap of Arc mRNA, allowing FMRP to join the complex, thus inhibiting Arc translation [103]. In summary, Arc seems to play a central role in the regulation of synapse plasticity and is endowed with a region with a peculiar structure, resembling the retroviral GAG domain. Through this domain, Arc may form capsid-like structures and attract RNAs for transport in neurons [66,184].

An example of the opportunities of regulation offered by local translation has been described in rat dorsal root ganglion (DRG) neurons and concerns the mRNA encoding Growth-Associated Protein 43 (GAP-43), a protein involved in axon elongation [185]. This messenger is modified by N^6^A methyltransferase (a ‘writer’), then transported along the axon in a translationally repressed state, and eventually translated only when methylation is removed by the Fat Mass and Obesity-associated protein (FTO), an m^6^A ‘eraser’ locally translated in axons [79]. There is also another mechanism regulating GAP-43 translation in rat axons: the KH-Type Splicing Regulatory Protein (KHSRP), an RBP able to inhibit GAP-43 translation, is itself inhibited by the non-coding axon-enriched lincRNA regulating axon elongation (ALAE) RNA. When ALAE interacts with KHSRP, this latter RBP cannot block GAP-43 mRNA translation anymore. Consequently, the absence of ALAE implies GAP-43 reduction and axon malfunctioning [186]. In both types of regulation, the region of the mRNA involved is the 3′-UTR [8,79,186].

Finally, two further examples of locally translated mRNAs concern rat Synaptosomal-Associated Protein of 25kDa (SNAP-25), a constituent of the SNARE complex, and β-catenin (a protein involved in the formation of a cell adhesion complex); both proteins are indeed synthesized in situ, allowing the formation of the presynaptic structure by the interaction with other proteins [187,188].

### 2.4. RBPs as Regulators of mRNA Pre-Localization in Brain Cells

As already mentioned, post-transcriptional regulation in the nervous system is based upon the activity of a set of binding proteins (RBPs) able to recognize specific signals and/or structures in the partner messengers [5,66,74,189]. The existence of some RBPs able to interact with different mRNAs to regulate a given function has been also demonstrated, as it is the case regarding the splicing factor poly-glutamine rich (SFPQ). This protein controls neurotrophin-dependent axon viability, by binding in the nuclei, cytoplasm, and axons of *Xenopus laevis* dorsal root ganglion sensory neurons, the mRNAs encoding the laminB2 (Lmnb2), a protein that is usually part of the nuclear lamina, and Bclw (an antiapoptotic protein), including both messengers in the same RNA granule, and transports them along the axons [190]. Bclw is involved in the local inhibition of the apoptosis following neurotrophin stimulation, therefore blocking axon degeneration [191]; Lmnb2 mRNA has been identified in axons, where it is translated by localized ribosomes, and the protein is involved in mitochondrial functions that support axon survival [192].

The activity of RBPs is most probably modulated by a cohort of enzymes which modify specific protein sites, by phosphorylation, methylation, ubiquitination, or SUMOylation [193]. Among RBPs, there are many different classes of factors playing specific roles in RNA metabolism. Of these groups, two are necessary for the RNAs to acquire their correct tridimensional structure: RNA chaperones, such as RNA helicases [194], and proteins that stabilize folded RNAs [195].

As discussed above, alternative polyadenylation is one of the regulation mechanisms in RNA metabolism; for example, RBPs, such as FUS and embryonic lethal abnormal visual system (ELAV)-like proteins, regulate the length of the 3′-UTR of a variety of brain-specific mRNAs [196,197]. In *Drosophila*, it has been shown that the process occurs in a peculiar way that directly connects the initiation of transcription with 3′-UTR elongation. ELAVs bind to the DNA promoter and to the 3′-UTR that is eventually transcribed, while the RNA polymerase II pauses at the initiation site. In this way, ELAVs impede the access to the proximal polyadenylation sites and sponsor the production of longer 3′-UTRs [198]. The 3′-UTR is also involved in the regulation of mRNA stability and localization by its binding to RBPs that recognize specific sequences/structures. As mentioned, proteins are arranged together in granules, thus many different RBPs, such as ZBP1, FMRP, or Staufen2, cooperate in all these functions [75,199]. Notably, many of these RBPs have a function at different steps of RNA maturation, transport, and localization. As discussed above, in order to send mRNAs to the neuronal periphery, anchoring of the RBPs to motor proteins is also required [200,201,202]. By reconstituting an mRNA transport complex formed by adenomatous polyposis coli (APC) RBP, the adaptor protein Kinesin-associated protein 3 (KAP3)n and Kinesin-2, what appears as the minimal requirement to transport mammalian mRNAs to the axons was demonstrated [203]. Moreover, APC binds the 3′-UTR of the mRNA, localized to microtubules in the periphery of the growth cone, of β2B-tubulin, a neuronal protein necessary for axon migration. Interfering with this interaction causes β2B-tubulin mRNA reduction and depletes dynamic microtubules at the periphery of the growth cone, thus blocking migration. A tempting suggestion coming from these results is that microtubules could affect the synthesis of their subunits in a self-organizing way [204]. A similar picture is offered by the RBP Nucleolin, which localizes the mammalian target of rapamycin (mTOR) mRNA to axons in order to sustain axon regeneration [205]. It is possible that the control of translation could be essential for the realization of certain processes such as axon regeneration; axon injury indeed stimulates protein synthesis, probably activating specific mRNAs by methylation in an ‘epitranscriptomic’ way [67].

An RBP with a fundamental role in the regulation of neuronal membrane excitability is Pumilio2 (Pum2), which regulates the local translation of mRNAs encoding sodium channels, such as the sodium voltage-gated channel alpha subunit 8 (Scn8a, encoding Nav1.6), to control neuronal excitation [206]. It has also been demonstrated that mRNAs which bear Pum2 recognition elements are retained in the neuron cell body during the initial stages of mammalian development. In this way, only selected RNAs are transported along neuronal extensions, while the translation of other mRNAs is impeded at the cell periphery. Eventually, but only when Pum2 expression is reduced, the same mRNAs are transported along the axon and translated in situ [207].

As mentioned above, during development, neuronal branches are elongated thanks to a set of proteins, among which are GAP-43, as well as β-actin. To get transported to the right place, their messengers are bound by zipcode-binding protein 1 (ZBP1) and included into an mRNP that travels along dendrites and axons. Unbound ZBP1 is involved in the induction of apoptosis but, when included in the GAP-43/β-actin complex, it is not, and neurite growth is allowed. The release of the mRNAs from the complex formed with ZBP1 is determined by specific axonal or dendritic extension signals that activate SRC kinase, which, in turn, phosphorylates ZBP1 [185,208]. Interestingly, β1-importin, a protein normally involved in nuclear import, has been recently described as a possible RBP because of its presence in neurites and its interaction with the Neuritin 1 (Nrn1) mRNA, encoding a protein which participates in neurite outgrowth [209]. The two molecules are included in a granule found at branching sites during neuronal differentiation that also contains Ras-GAP SH3 domain-binding protein 1 (G3BP1), an enzyme of the Ras signal transduction pathway [210].

Ribosomes were usually regarded as invariant organelles, formed by two subunits, i.e., small 40S and large 60S, and containing the same set of proteins in all tissues. Recently, this notion has been challenged by the finding that ribosome proteins necessary for the translation of certain mRNAs may vary in different tissues [211,212]. In mouse embryos, it was demonstrated by mass spectrometry that some ribosomal proteins (RPs) of both the large (RPL) and the small subunit (RPS) were differentially represented in polysomes and in free subunits (i.e., RPL10A, RPL38, RPS7, and RPS25) [213]. Moreover, in mouse embryos, a mutation of the RPL38 has been identified that causes distinct defects, among which specific variations of the axial skeleton, due to the alteration of the translation of a group of homeobox messengers. RPL38 seems to allow the inclusion of these mRNAs in the 80S subunit, providing a specific translational control [212,214]. In addition, RPL13a was shown to control the translation of the Ceruloplasmin (CP) mRNA by specifically binding to it. In response to interferon gamma stimulation, RPL13a is phosphorylated and released from the ribosome and binds the interferon Gamma-Activated Inhibitor of Translation (GAIT) element in CP mRNA, thus inhibiting its translation [215].

### 2.5. RBPs and Neuronal Plasticity

Both motor and cognitive learning and memory processes, and, therefore, adaptation to environmental conditions, depend on neuronal plasticity. Under physiological conditions, adult neuronal plasticity mainly concerns the synapses. Experience indeed induces a strengthening or weakening of the nerve impulse transmission efficacy.

Synaptic plasticity does not depend only on the activity of nerve cells but also on glial cells that, by releasing a variety of molecules, influence neuronal transmission (reviewed in [216]).

These changes involve gene activation, but also changes in both the presynaptic and postsynaptic localized synthesis of proteins. The fine regulation of these processes depends on an efficient coordination of mRNA transport and metabolism, mostly managed by RBPs that, as discussed above, bind mRNAs and regulate their transport, stability, and translation, thus controlling proteostasis in response to synaptic activity [189]; aberrant translation could indeed affect synaptic plasticity and lead to neurodevelopmental disorders, such as autism spectrum disorders (ASDs) [217,218], and neurological pathologies, as well as neuropsychiatric disorders in adults, such as amyotrophic lateral sclerosis (ALS) [189,219].

Given the importance of continuously regulating proteostasis, at the level of nerve endings, and the high level of polarization of nerve cells, a particularly active and functional cytoskeleton is required that acts as a complex of tracks on which both organelles and RNA–protein complexes can travel from the cell body to the periphery and vice versa, thanks to kinesin and dynein cell motors, respectively [216]. Then, in the periphery, microtubule-carried cargoes are transferred to actin filaments and their associated motor myosin [220]. As expected from these considerations, an intact cytoskeleton is essential for the learning and memory processes [221,222,223,224]; moreover, it has been suggested that both microtubules and microfilaments with their own charges can bind ions and even affect electrical signals travelling along neuronal processes [223].

While the presence of a protein synthesis system in the dendritic compartment has been recognized for a long time [225], the existence of similar activities has only recently been accepted for the axonal periphery, on the basis of convincing evidence of an axonal and presynaptic protein synthesis system [226].

As discussed above, it is now widely accepted that mRNAs are transported to the cell periphery in a silent state, due to interactions with other regulatory RNAs and with RBPs. At the level of the synapses, signals related to the neuronal activity itself elicit modifications of the inhibiting molecules (for example, phosphorylation of some RBPs), thus allowing mRNA translation and accumulation of new proteins; some of the newly synthesized proteins might even come back to the cell body, where they can induce the modification of the chromatin structure and gene expression.

Among the RBPs involved in learning and memory consolidation, there is the growth arrest and DNA damage-inducible protein 45 alpha (GADD45α), which regulates the stability of transcripts by binding to their 3′-UTR. Mice deficient in the GADD45α gene show reduced levels of memory-related mRNAs and problems in learning and long-term memory potentiation [227].

Other factors that, in addition to a clear role in brain development, also influence memory processes are Staufen proteins, of which only one has been found in invertebrates, while in vertebrates, two proteins, with different distribution, are known: Stau1 and Stau2 [5,228,229]. Stau2 is largely expressed in the brain [230]. However, by using a siRNA-based methodology for silencing Stau1 in hippocampal pyramidal neurons, it has been found that long-term potentiation (LTP) is specifically affected, but not early LTP [231]. On the other hand, Stau2 is able to regulate the balance between LTP and Long-Term Depression (LTD) [66,232]. Notably, with similar experiments, it has also been found that the RNA-binding domain 3 of the protein seems to have a fundamental role in driving dendritic arborization and synapse formation [233].

The immunostaining of Stau proteins in hippocampal neurons indicated a mainly somatodendritic localization [234], in association with RNA granules [235]; interestingly, the two proteins seem to be present in different granules [5]. In any case, Stau association with granules and its transport to dendrites involve functional microtubules [109,235]. Although mostly present in the cytoplasm, Stau proteins are also present in the nucleus [5].

Another important protein involved in memory processes is FMRP, one of the first RBPs discovered and probably the most studied [3]; its depletion causes enhanced LTD and reduced LTP. Recently, it has also been reported that both *Drosophila* and human FMRP stimulate protein kinase A (PKA), in relation to learning and memory [236].

A very important role in stabilizing mRNAs during learning and memory is also played by the proteins of the ELAV family [6]. Among these, HuD, which, in addition to the canonical mRNA binding functions, is able to bind and regulate non-coding RNAs, among which circRNAs, which are also involved in learning and memory [133]. Of particular importance seems to be the ability of HuD to bind mRNAs such as those encoding a few fundamental regulators of learning/memory, among which BDNF, CaMKIIα, and the Homer protein homolog 1a (Homer1a) [237,238].

Interestingly, some already mentioned RNA modifications can also be important for recognizing and binding RBPs, which then control their translation. One example has been recently given for the N^6^-methyladenosine (m^6^A), which, by specifically binding to the protein known as the YTH domain-containing family protein 1 (YTHDF1), induces the translation of the modified mRNAs in the adult hippocampus, in response to neuronal activity, thus probably stimulating the formation of memories [239].

The CPEB influences neuronal plasticity, and hence learning and memory, too [240]. Interestingly, this protein rests on some prion-like properties for its functions [241,242]. Notably, a feedback loop seems to exist involving CPEB and CAMKIIα, where phosphorylation of CPEB by CAMKIIα reduces the inhibition of CAMKIIα mRNA translation by the CPEB [243]. By the interaction with motor proteins, the CPEB also participates in the transport of the mRNAs under its translational control, such as those encoding CAMKIIα and Map2, to dendrites [112].

Notably, as discussed below, both neurons and glial cells are able to release extracellular vesicles (EVs), and it is possible to suppose that at least some of the molecules involved in neuroplasticity and learning/memory processes are secreted via EVs (reviewed in [216]). Notably, indeed, EVs contain proteins, lipids, and different classes of RNA, all able to modify the genetic activity of the recipient cells [244].

## 3. Role of RBPs in RNA Sorting to Extracellular Vesicles

Extracellular vesicles (EVs), considered for a long time a way used by cells to discard unwanted materials, have now been definitively recognized as a central way for cell-to-cell communication and molecule exchange, both in eukaryotes and in prokaryotes, as well as for trans-kingdom communications [216,245,246,247,248,249,250,251,252,253,254,255,256,257,258,259], thus suggesting an ancient evolutionary origin of EVs.

Although EV production is particularly evident in tumor cells, all tissues of multicellular organisms seem to be able to release EVs, and this ability is probably related to the intercellular crosstalk that allows functional integration within each tissue, and also response integration within the organism as a whole. EVs indeed contain a variety of molecules, such as proteins, coding and non-coding RNAs, and lipids and can also deliver small compounds of metabolic origin, such as lactate [248]. Notably, most of these molecules are enriched in EVs compared with the whole cell content. Thus, one central question concerns the mechanisms responsible for their specific sorting to EVs.

### 3.1. Extracellular Vesicles (EVs): Origin and General Functions

Although, once released, some EVs probably blow up in the extracellular matrix, thus releasing their content outside the cells [216,260], most of their cargo enters the surrounding cells through different internalization mechanisms, among which either clathrin-dependent or clathrin-independent endocytosis, phagocytosis, lipid rafts-mediated processes, and direct EV fusion with neighboring cell membranes [216,260,261,262]. Sometimes, the material acquired in such a way by the cells can be released outside again through a kind of transcytosis, and this mechanism might be of special importance at the level of the blood–brain barrier (BBB) [263]. Independent of the specific mechanism used to enter surrounding cells, once inside, EV cargo can induce epigenetic modifications of the receiving cells (i.e., horizontal transfer of cellular properties); as mentioned, EVs contain, indeed, different classes of RNAs. Among these incoming nucleic acids, mRNAs can be translated, miRNAs can repress translation of endogenous messengers, while lncRNAs/circRNAs can sponge endogenous miRNAs, thus allowing the enhancement of the translation/stability of some resident mRNAs. Recently, we also proposed that transcription factors with the ability of binding both DNA and RNA can find their way to neighboring cells by binding to some RNAs transported by EVs; once in a new cell, however, they might bind DNA, thus inducing a modification of gene transcription [264]. Intriguingly, it has also been reported that EVs can transport many miRNA passenger strands (i.e., the apparently non-functional strands of the duplex miRNAs; only the so-called guide strands are, indeed, transferred to the abovementioned RISC complexes and can pair with the target mRNAs) [265,266,267]. The presence of passenger miRNA strands in EVs can have one or both of the following explanations: (i) cells discard the useless strands via EVs, and (ii) passenger strands do have a function and their transfer to other cells via EVs is part of an epigenetic action of EVs themselves.

On the basis of their cellular origin, EVs have been classified as: (i) microvesicles/ectosomes (MVs; 100–1000 nm), formed through a cell membrane budding process, resembling virus release from cells; (ii) exosomes (30–100 nm), which derive from components of the endosomal compartment known as multivesicular bodies (MVBs); and (iii) apoptotic bodies, which have, however, completely different properties as they derive from the breakup of dying cells [248,250,268,269]. Apoptotic bodies contain, for example, high amounts of condensed and fragmented nuclear DNA. Even if composition differences have been described, in addition to differences in size, it is not yet easy to distinguish ectosomes and exosomes [249,270]. Thus, herein, we refer to a general population of EVs.

Interestingly, the composition of EVs also varies depending on the producing cells, thus suggesting the possibility to use EVs, collected from different biological fluids, as diagnostic biomarkers [271,272,273,274,275].

### 3.2. Roles of EVs in the Nervous System

All the cells of the Nervous System normally release EVs, probably both exosomes and ectosomes [216,248]. Among the proteins present in EVs released from neurons, for example, angiogenic factors, such as vascular endothelial growth factor (VEGF) and fibroblast growth factor 2 (FGF2), which can stimulate BBB formation and/or maintenance, have been found [276], together with some isoforms of glutamate receptor subunits [277], the L1 cell adhesion molecule (L1CAM) [277], and even the glycosylphosphatodyl-inositol(GPI)-anchored prion protein [277]. Notably, EVs released by neurons might also act as a trans-synaptic way of communication [278,279], giving rise to both the potentiation of the traditional “wiring” (synaptic) transmission and to what has been called, since the eighties, non-synaptic “volume” transmission [216,280,281,282].

Like neurons, astrocytes also release a variety of proteins via EVs, among which VEGF and FGF2 [283], matrix metalloproteinases (MMPs) [284], Hsp70/Hsc70 [285], apoliprotein D (ApoD) [286], and glutamate transporters [287]. On the other hand, oligodendrocytes include in their EVs, in particular during nervous system development, among other molecules, myelin components, such as the myelin basic protein (MBP) [288], and also glycolytic enzymes [289]. Moreover, it has been found that oligodendrocytes of the central nervous system (CNS), as well as Schwann cells (SCs) of the peripheral nervous system (PNS), can even transfer ribosomes to neuronal axons [290,291]. Given this ability of SCs, as well as of oligodendrocytes, to release EVs that allow the lateral transfer of molecules to axons [292,293,294], it can be suggested that EVs are also involved in the transport to axons of ribosomes, which can then allow the local translation of pre-localized mRNAs, in response to specific extracellular signals.

Finally, microglial cells also release EVs, which can contain proteins able to regulate inflammatory processes, such as interleukin-1β (IL-1β) [295,296].

As already discussed, EVs also contain different classes of RNAs, among which a variety of miRNAs, which also contribute to the epigenetic modification of the receiving cell activities (reviewed in [216]). As a whole, the EV-dependent events certainly allow the reciprocal regulation of brain cell functions. In addition, the controlled release of EVs and their cargoes has important effects on synaptic plasticity and, consequently, also on learning and memory processes [216].

However, the described basal ability of all brain cell types to release EVs that contain a variety of cargo molecules becomes a double-edged weapon in pathological conditions: as discussed below, indeed, in most nervous system pathologies, EVs are still produced and in most cases they contain altered molecules (for examples, prions or other protein aggregates) [268,297,298,299,300,301,302,303,304] that are transferred to neighboring cells, thus spreading the pathological molecules and conditions from cell to cell, like an infection.

### 3.3. Specific Sorting of Molecules to Nascent EVs: Possible Role of RNA-Binding Proteins

As mentioned, a special question concerns the mechanisms that allow the specific loading of given RNAs and proteins to EVs. It seems that some membrane lipid components have a central role in sorting both proteins and RNAs to EVs [266,305]. From this point of view, lipid rafts (LRs) seem to have a special importance: these microdomains of the membranes are indeed enriched in ceramide, which has two important properties: (i) it is cone-shaped, and thus can induce curvature of the membrane [266], and (ii) it can be hydrogen-bonded to itself [266,306], and probably also to proteins [307,308]. These ceramide properties can facilitate the formation of EVs [308,309,310], on the one hand, and the interaction with specific proteins that are thus bound to the membrane domains involved in EV formation; for example, it has been reported that the RBP known as Heterogeneous Nuclear Ribonucleoprotein A2/B1 (HnRNPA2B1) has affinity to ceramide and is released into EVs. The association with HnRNPA2B1 can allow, in turn, the sorting of a class of miRNAs found enriched in exosomes and thus named EXOmiRNAs [266,311,312], but also of other classes of RNA, such as LncRNAs [313]. As a further demonstration of ceramide involvement in EV formation and composition, it has been found that hyper-activation of neutral sphingomyelinase 2 (nSMase2), the enzyme that hydrolyzes sphingomyelin and releases ceramide, is also implicated in the increase in EVs in tumors [314], as well as in neuropathologies [315,316]. It is also worth underlining that many proteins are post-translationally modified in order to be sorted to EVs; for example, the just-mentioned HnRBPA2B1 present in EVs has a molecular mass higher than that of the same protein inside the cell, and this difference is due to the sumoylation of the sorted protein [311,317,318,319]. Interestingly, α-synuclein, a fundamental protein of synapses, also involved in Parkinson’s Disease (PD), is similarly sumoylated to be sorted to EVs in pathological conditions [320]. Like α-synuclein, Tau protein, the axonal microtubule-associated protein that, when hyperphosphorylated, forms aggregates responsible for most problems associated with Alzheimer’s Disease (AD), can be sorted to EVs when phosphorylated at Thr-1801 [318,321]. Coming back to RBPs, HnRNPA1 is sumoylated as well and, after recognizing a GAGAGA motif present in the 3′-region of some miRNAs, can sort them to EVs [318]. In addition, some membrane proteins, such as Caveolin-1, can also contribute to sorting by interacting with RBPs [319,322]. From this point of view, an interesting finding concerns the involvement, in RNA trafficking, of some phospholipid-binding membrane proteins known as annexins and, in particular, of Annexin 2 (ANXA2) [323]. These proteins can be modified by phosphorylation, acetylation, and S-glutathionylation; interestingly, these post-translational modifications can modulate the interaction with other proteins and with both coding and non-coding RNAs [324]. Of note, it has also been found that the autophagy process, traditionally considered a major lysosome-dependent intracellular degradation pathway, is also involved in secretion and, in particular, in EV-dependent secretion [325]. By proteomic and RNA-profiling analyses, it was found that a variety of RBPs and RNAs require the Microtubule-associated protein 1A/1B-light-chain 3(LC3) conjugation machinery to be loaded into EVs and, again, the action of nSMase2 [325]. Two RBPs seem, in particular, to regulate the non-coding RNA enrichment of EVs via secretory autophagy: the scaffold attachment factor B1 (SAFB1) and HnRNPK [326].

On the other hand, it has been also proposed that RNAs themselves can interact with ceramide-rich membrane domains through a variety of motifs, each of which shows different degrees of affinity for the lipid [266]. Moreover, hydrophobic modifications, such as methylation and isopentenylation, could further increase the ability of RNAs to interact with the lipid raft-containing membrane domains [266].

Many studies have, in any case, confirmed the involvement of RBPs in sorting RNAs into EVs [324]. By recognizing and binding specific sequences/structures of RNAs, RBPs are not only responsible for all the steps of RNA metabolism, but also for loading them into EVs. Besides the mentioned HnRNPA2B1 and HnRNPA1, other members of the HnRNP family of RBPs have been reported to control RNA sorting of their target RNAs; among them, for example, are included HnRNPQ, also known as Synaptotagmin-binding, Cytoplasmic RNA-Interacting Protein (SYNCRIP) [319,327], and HnRNPK [328].

Many other RBPs, belonging to a variety of families, have also been found to contribute to RNA sorting to EVs. Shurtleff et al. [329], for example, reported the involvement of the RBP known as Y box containing 1 (YBX1). This latter protein, the name of which refers to the presence in its structure of a domain able to recognize the so-called Y box motif (CTGATTGGCCAA) on DNA, also contains RNA-binding motifs, among which the so-called cold-shock domain (CSD), and can interact with both DNA and RNA, thus being part of different complexes, containing both other DNA-binding and RNA-binding proteins [324].

Another protein with a variety of roles in RNA metabolism is Human Antigen R (HuR), belonging to the RBP family of embryonic lethal abnormal vision (ELAV) proteins. HuR has also been recognized as a sorting factor for some miRNAs [330].

Among RBPs with a role in the nervous system and found in EVs, a special interest has been paid to proteins such as FUS and TDP-43, both of which are altered in ALS, a fatal neurodegenerative disease due to selective loss of motor neurons of the spinal cord. These proteins are normally able to shuttle between the nucleus and the cytoplasm, and are involved in many steps of RNA metabolism, from maturation to transport. As discussed below, when altered, they aggregate and are mislocalized to the cytoplasm, from where they also enter EVs [5,331], thus becoming able to be transferred to other cells with a prion-like mechanism.

Of course, the processes that allow EV secretion also require modification of the cell shape; this means that it also depends on interaction among cytoskeletal proteins, such as actin and myosin, with also a requirement for ATP hydrolysis, and very often for an increase in cytosolic calcium [186].

As a final comment concerning the specificity of sorting, it is worth noting that polarized cells might produce EVs with different contents and properties, depending on their origin from basal or apical membrane domains [332,333]. This consideration is of particular importance for neurons, which are the most polarized cells of the organism, but probably also for all the classes of glial cells.

## 4. RBPs and Neurological Diseases

Given the fundamental role played by RBPs in neural development and in controlling complex brain functions such as learning and memory, it is not surprising that RBP dysfunctions are involved in a variety of nervous system pathologies. As discussed above, RBPs actually regulate all the steps of mRNA maturation, trafficking and translation; thus, any alteration in their nucleocytoplasmic localization, as well as mutations that affect their interaction with RNA or with other proteins, can have large effects on nerve cell physiology. Below, we discuss a few examples of neurodegenerative diseases for which involvement of RBPs has been demonstrated or suggested on the basis of the existing data.

### 4.1. Amyotrophic Lateral Sclerosis (ALS)

Many of the genetic mutations found in cases of both familial and sporadic ALS involve genes encoding RBPs, such as TDP-43 and FUS. Alteration of the nucleocytoplasmic trafficking of these proteins is a well-defined pathological feature of ALS and leads, as expected, to impaired regulation of RNA metabolism within cells.

#### 4.1.1. TDP-43

TDP-43 is a DNA-/RNA-binding protein of 414 amino acids, predominantly present in the nucleus. Its main function seems to be the regulation of different aspects of RNA metabolism, including splicing, but also stress granule formation, and transport and protection of many RNAs [334]; these functions are mediated by its preferential binding to the UG-rich regions in the long introns of pre-mRNA [335]. The protein structure is composed by: (i) an N-terminal domain (NTD) (aa 1–102), which includes the nuclear export signal (NES), (ii) an intermediate region with two RNA recognition motifs (RRM1 and RRM2) and a nuclear localization sequence (NLS), and (iii) a C-terminal domain (CTD) (aa 274–414), within which there are a glutamine/asparagine-rich (Q/N) domain (aa 345–366) and a glycine-rich sequence, essential for protein–protein interactions (aa 366–414) [336].

The pathogenicity of TDP-43 in ALS appears to be due both to loss of nuclear functions and to gain of cytoplasmic functions. The mechanisms of TDP-43 loss of functions have been studied by analyzing the effects of its deletion on RNA metabolism. For example, depletion, by antisense oligonucleotides, of TDP-43 from adult mouse brain results in RNA mis-splicing; in particular, TDP-43 depletion affects the production of a variety of transcripts, among which those for FUS and Progranulin, which also have a specific role in ALS [335].

Other evidence suggests a strong contribution of gain of cytoplasmic toxicity in TDP-43-related ALS. First of all, when present at high concentrations, TDP-43 aggregates into insoluble cytoplasmic inclusions, which have been characteristically found in post-mortem motor neurons from ALS patients [337]. The TDP-43 CTD is particularly prone to aggregation, probably because it contains the Q/N region that has prion-like properties [338,339]. In addition, indeed, most of the TDP-43 mutations leading to the disease were found in the Q/N domain [340]. A second observation concerns the fact that TDP-43 cytotoxicity depends on its cleavage [341], even if both full-length and truncated forms of TDP-43 can be found in ALS aggregates [336]. In particular, the most pathogenic forms seem to be fragments of 25–35 kDa, commonly present in the intra-cytoplasmic deposits that result from the aberrant activity of some caspases [342]. Interestingly, the aggregation of these TDP-43 C-terminal fragments seems to be at least in part reversible [343].

More recently, it has been observed that cell death triggered by full-length TDP-43 occurs prior to protein fragmentation by caspases [341]. Moreover, it has also been reported that, although they are a neuropathological feature of ALS molecular pathobiology, the TDP-43 C-terminal fragments (CTFs) are probably not the primary cause of ALS [344]. In any case, clearance of both full-length and truncated TDP-43 proteins has consequences on protein pathology. For example, mutations of Ubiquilin-2 disrupt the ubiquitin-proteasomal degradation of TDP-43 and enhances its aggregation [345]. Intracellular deposition of TDP-43 can occur within the soma, as well as in axons and dendrites, with alterations in the transport of many mRNAs [346].

On the other hand, some authors believe that RNA binding to TDP-43 is protective for protein aggregation [347,348], and it seems, indeed, that the prolonged lack of interaction with RNA favors the aggregation of the mislocalized proteins in the cytoplasm [348].

#### 4.1.2. FUS

FUS (also called translocated in liposarcoma, TLS) is an RBP of 526 amino acids that contains an N-terminal serine–tyrosine–glycine–glutamine (SYGQ)-rich domain, a Gly-rich domain, an RRM, multiple Arg-Gly-Gly repeats, a zinc finger motif, and a highly conserved C-terminus, which encodes for an NLS that is recognized by a complex of import receptors, including Transportin 1 [349,350,351]. The functions of FUS are not yet clearly known, but it appears that the protein participates in the processing of numerous RNAs and microRNAs, and its binding sites on target RNAs are probably generally larger than those for TDP-43 [352]. Like TDP-43, FUS is present in the cytosolic aggregates of affected motor neurons in ALS. Although the main traits of ALS pathobiology are similar for FUS and TDP-43 alteration, TDP-43 anomalies are absent in FUS-related ALS patients.

Interestingly, mice with FUS-related mutations or with overexpression of wild-type FUS develop neurodegeneration of motor neurons in a way similar to what happens in ALS [353]. Like TDP-43, FUS is normally present both in the nucleus and in the cytoplasm, and continuously shuttles between the two compartments; however, its physiological localization is predominantly nuclear, and the accumulation of FUS in the cytoplasm is pathological [354]. Cytoplasmic accumulation of predominantly nuclear FUS is often due to mutations in its C-terminal region that disrupt the NLS, causing a reduced ability of FUS to enter the nucleus [355].

FUS-dependent pathology in ALS is, however, explained by both loss of function and gain of toxicity mechanisms. The latter was first highlighted by the group of Shelkovnikova and colleagues. These authors developed a truncated form of FUS (FUS 1-359) by depriving the protein of the RNA recognition domain, in order to evaluate the aggregative effects independently of the regulatory functions of RNA metabolism. Mice transgenic for FUS 1-359 developed FUS-positive aggregates in the cytoplasm, sufficient to reproduce clinical ALS phenotypes [356]. On the other hand, according to a pathogenetic mechanism of ALS supported by the loss of function of FUS, Lagier-Tourenne and colleagues showed that depletion of FUS from adult mouse and human brain caused dysregulation of more than 600 RNA and altered the splicing of more than 350 of them. Moreover, after FUS or TDP-43 depletion, some of these RNAs reduced their expression levels in stem cell-derived human neurons and in TDP-43 inclusions in motor neurons of patients with ALS [352].

### 4.2. Multiple Sclerosis (MS)

Multiple sclerosis (MS) is an autoimmune demyelinating disease of the central nervous system (CNS), in which neurodegeneration plays an important role, especially during the progression phase [357]. Neuronal and axonal degeneration is already present during the acute inflammatory attack, but the continuous chronic inflammation is the real cause at the basis of the neurodegenerative processes in MS [358]. The pathology is characterized by demyelination and significant loss of neurons in the cortical areas [359], but also in deep gray matter nuclei [360]. Moreover, other studies show that axonal degeneration is also present in the early stages of MS, and that this process contributes to the accumulation of disability [361,362]. The molecular mechanisms of neurodegeneration are currently unknown. Recently, it has been hypothesized that RBP dysfunction may play a role in MS as in other neurological diseases such as ALS and fronto-temporal dementia (FTD) [363].

Normal-appearing cortical neurons from MS patients show increased cytoplasmic localization and decreased nuclear localization of both TDP-43 and HnRNPA1 [364,365]. In active demyelinating lesions of MS patients, TDP-43 is mislocalized to the cytoplasm of oligodendrocytes. In the same lesions, FUS distribution is normal [366]. In an experimental autoimmune encephalomyelitis (EAE) model of MS, mislocation of these RBPs is also noticed in the gray matter of the spinal cord and correlates with neuronal loss and with a local increase in neurodegeneration markers [364]. Moreover, antibodies against HnRNPA1 worsen the clinical course of EAE and cause widespread neurodegeneration, especially in the ventral spinocerebellar tract and deep white matter of the cerebellum [367].

Another RBP of particular interest in MS seems to be HuR. This latter protein is overexpressed in the cytoplasm of microglia and in the spinal cord of EAE mice; most importantly, the intrathecal administration of anti-HuR antisense oligonucleotide reduces neuroinflammation and lymphocyte infiltration [368]. Moreover, HuR promotes differentiation of T helper 17 cells (Th17) [369], where it regulates the expression of CCR6, the C-C chemokine receptor 6 (CCR6), thus promoting EAE [370]. HuR-Knockout CD4+ T cells are less efficient in inducing EAE [369]. Therefore, due to the supporting role of the neuroinflammatory processes, HuR appears to be a target of particular interest for modulating the course of MS from its onset. Moreover, MS patients often suffer from nociceptive pain, and the appearance of this symptom correlates with a more unfavorable clinical course. An increased HuR expression was demonstrated in the spinal cord of EAE mice with a hypernociceptive behavior, and the silencing of HuR not only improved painful symptoms, but also reduced motor dysfunction and the severity of demyelination [371]. Although different authors confirm an increased expression of HuR in the CNS of EAE models [368,369], other authors found reduced levels of HuR in the peripheral blood mononuclear cells (PBMC) from 52 MS patients. The reduction in HuR in the PBMCs correlates with an increase in clinical disability. Moreover, they report the loss of the interaction of HuR with one of its targets, the mRNA encoding HSP70-2, a protein with a probable role in the activation of the immune system, the increased expression of which is associated with a greater risk of MS [372].

Therefore, it appears that the alterations caused by RBP disfunction in MS could result either from their incorrect nucleocytoplasmic localization, as in the case of TDP-43 and HnRNPA1, or from a change in their expression levels, as in the case of HuR.

### 4.3. Alzheimer’s Disease (AD)

Alzheimer’s disease (AD) is a neurodegenerative disease characterized, at the clinical level, by the development of a progressive form of dementia and, at the cellular level, by the formation of both intracellular neurofibrillary tangles and extracellular deposits of beta-amyloid [373]. The intracellular tangles are mainly formed by Tau, a MAP involved in cytoskeletal stabilization and axonal transport [374]. The affinity of Tau for microtubules mainly depends on its state of phosphorylation, with hyperphosphorylation causing enhanced detachment from tubulin [375]. In AD, Tau is hyperphosphorylated and, therefore, binds with less affinity to tubulin; as a result, microtubules tend to be disassembled with the consequent alteration of axonal transport [376]. The C-terminal end of tubulin molecules is acidic, and the tau–tubulin interaction is based on the interaction between a polycation (Tau) and a polyanion (Tubulin). It is thus possible that, in a similar manner, under certain conditions, Tau is able to interact with other polyanionic molecules such as RNAs [377]. In particular, it has been reported that RNA can cause the conversion in vitro of soluble Tau into the paired helical filaments, the pathological fibrous assembly of Tau typical of AD [377]. Actually, Tau is probably able to bind RNA through the proline-rich and the microtubule-binding domains [378]. The coexistence of different kinds of polyanions around Tau in the cell probably causes crowding and polyanion-induced Tau condensation; it has been recently reported that this is indeed the case. Moreover, RNA and tubulin seem to compete for binding to Tau in these conditions [379].

In cells from mice brain and patients with AD, but also with FTD and corticobasal degeneration, both nuclear and cytosolic RNA–Tau complexes are enriched for small nuclear RNAs and small nucleolar RNAs (snoRNAs). Moreover, Tau aggregates alter pre-mRNA splicing by inducing miscolocalization to the cytosol of nuclear speckles, dynamic structures with a role in pre-mRNA splicing [374]. Actually, based on atomic force microscopy, Western blot, and immunoprecipitation experiments, it has also been reported that the well-known RBP Musashi forms oligomers in vivo; moreover, it increases in AD and, most importantly, it enters large assemblies that also contain Tau [380]. Moreover, the formation of these Musashi/Tau complexes seems to affect both nuclear functions (i.e., chromatin modifications and nuclear lamina assembly) and nuclear-cytoplasmic transports [381]. Interestingly, HNRNPA1 has also been found to associate with phosphorylated Tau in AD [382].

All these observations suggest that the Tau protein may also play a role in the pathogenesis of AD because of its ability to interact with RNA and/or with RBPs, thus causing a variety of errors at many levels in RNA metabolism. Further studies are, however, necessary to confirm whether these interactions actually exist in the cells, and to understand the molecular mechanisms on which they are based.

A brief summary of data concerning the demonstrated/suggested role of RBPs in three neurological diseases is given in Table 1.

## 5. Conclusions and Future Perspectives

The central role played by RBPs in controlling the physiology of both neurons and glial cells, both in development and in the adult nervous system, is by now widely accepted, as it is too their impact on all the steps of mRNA maturation, trafficking, stability, and translation (Figure 2). By allowing the correct localization of their target transcripts and their translation at the right moment, in response to specific signals, they indeed control the formation of the neuronal/glial networks in development, and also allow the establishment of higher functions, such as learning and memory. Moreover, it is also clear that RBPs can also interact with non-coding RNAs, thus mediating most of the functional RNA–RNA interactions in the cells.

Interestingly, most RBP functions somehow rely on the presence in their molecule of intrinsically disordered regions (prion-like domains), probably involved in the formation of membrane-less structures; such regions, of central importance for the formation of a variety of granules that contain proteins and different classes of RNAs, seem, however, to be also prone to form aggregates, as observed in many neurological pathologies. Further studies are thus necessary to completely understand the mechanisms that can transform a useful plastic structure into an aggregation-prone one.

Finally, as discussed in this review, RBPs are also able to enter extracellular vesicles (EVs) and are probably essential for the specific sorting of RNAs to them. Intriguingly, they can do that as part of both normal and aggregated complexes; as a consequence, when they reach surrounding cells, they can also contribute to propagating pathological states from one cell to the other ones. Thus, perhaps, pathology-specific RBPs present in circulating EVs, in the normal or in the aggregated form, might also be used as biomarkers of the pathologies themselves.

## Figures and Tables

**Table 1:** RBPs with a suggested role in some neurodegenerative pathologies.

Neurological Disease	RBPs	Predominant Localization in Normal Conditions	Suggested Functions	Tendency to Form Aggregates
Amyotrophic Lateral Sclerosis (SLA)	**TDP-43** [334,335,336,337,338,339,340,341,342,343,344,345,346,347,348] **FUS** [349,350,351,352,353,354,355,356]	Nuclear (neurons) Nuclear (neurons)	-splicing -granule formation -RNA transport and protection [334,335,336,337,346] Processing of different RNAs (among which miRNAs) [352,354]	yes [336,337,338,339,340,341,342,346] RNA binding seems to be protective for aggregation [348] yes [356]
Multiple Sclerosis (MS)	**TDP-43** [364,365,366] **HnRNPA1** [364,365,367] **HuR**	Nuclear (neurons) Nuclear (neurons) Cytoplasm (microglial cells) [368]	see above -different aspects of RNA metabolism [331] -miRNA sorting to EVs [318]	see above yes [383] observed in gliomas [384]
Alzheimer’s Disease (AD)	**Tau**	neuronal axons	Main function: regulation of microtubules dynamics [374] Hypothesized functions: -RNA binding [378,379], with a possible effect on splicing [374]	yes Tau has been reported to form complexes with some RBPs, such as Musashi [381] and HnRNPA1 [382] **NOTE**: RNA can cause conversion in vitro of soluble Tau into paired helical filaments [377]
